# β2-Adrenergic Receptor Expression and Intracellular Signaling in B Cells Are Highly Dynamic during Collagen-Induced Arthritis

**DOI:** 10.3390/biomedicines10081950

**Published:** 2022-08-11

**Authors:** Nadine Honke, Clemens J. Wiest, Georg Pongratz

**Affiliations:** 1 Department of Rheumatology, Hiller Research Center Rheumatology, University Hospital Düsseldorf, 40225 Düsseldorf, Germany; 2 Department of Internal Medicine II, University Hospital Regensburg, 93053 Regensburg, Germany; 3 Center for Rheumatologic Rehabilitation, Asklepios Clinic, 93077 Bad Abbach, Germany; 4 Medical Faculty of the University of Regensburg, 93053 Regensburg, Germany

**Keywords:** B cells, rheumatoid arthritis, IL-10, β2-adrenergic receptors, autoimmune disease, CIA mouse model, signaling pathway, p38, CREB

## Abstract

The sympathetic nervous system (SNS) has either a pro-inflammatory or anti-inflammatory effect, depending on the stage of arthritis. In the past, treatment of arthritic B cells with a β2-adrenergic receptor (β2-ADR) agonist has been shown to attenuate arthritis. In this study, the expression and signaling of β2-ADR in B cells during collagen-induced arthritis (CIA) were investigated to provide an explanation of why only B cells from arthritic mice are able to improve CIA. Splenic B cells were isolated via magnetic-activated cell sorting (MACS). Adrenergic receptors on B cells and intracellular β2-ADR downstream molecules (G protein-coupled receptor kinase 2 (GRK-2), β-Arrestin 2, p38 MAPK, extracellular signal-regulated kinase 1/2 (ERK1/2) and cAMP response element-binding protein (CREB)) were analyzed at different time points in naïve and arthritic B cells with and without stimulation of β2-ADR agonist terbutaline by flow cytometry. β2-ADR-expressing B cells increase during CIA without a change in receptor density. Moreover, we observed a profound downregulation of GRK-2 shortly after induction of arthritis and an increase in β-Arrestin 2 only at late stage of arthritis. The second messengers studied (p38, ERK1/2 and CREB) followed a biphasic course, characterized by a reduction at onset and an increase in established arthritis. Stimulation of CIA B cells with the β-ADR agonist terbutaline increased pp38 MAPK independent of the timepoint, while pERK1/2 and pCREB were enhanced only in the late phase of arthritis. The phosphorylation of p38 MAPK, ERK1/2 and CREB in the late phase of arthritis was associated with increased IL-10 produced by B10 cells. The change of β2-ADR expression and signaling during sustained inflammation might be an integral part of the switch from pro- to anti-inflammatory action of sympathetic mechanisms in late arthritis.

## 1. Introduction

Rheumatoid arthritis (RA) is an autoimmune disease characterized by chronic joint inflammation and finally associated with joint destruction [1]. Sympathetic tone is dominant in both RA patients and RA animal models, suggesting involvement of the sympathetic nervous system (SNS) in the disease process [2,3,4]. Moreover, several studies demonstrated that the SNS directly influence the course of chronic joint inflammation [5,6,7]. In both, RA and collagen-induced arthritis (CIA), a common mouse model, to study RA, tyrosine hydroxylase-positive cells (TH+) are present in the inflamed synovium [8]. These cells are capable to provide local sympathetic transmitters [9]. In addition to TH+ cells, sympathetic nerve fibers are also found in the synovial tissue and are modulated by specific, nerve repellent factors during RA [6,7].

The SNS is able to affect immune cells and their cytokine production [10,11]. This SNS-dependent influence on immune cells is due to 1. the innervation of sympathetic nerve fibers in lymphatic organs and their close proximity to local immune cells [12,13], and 2. the ability of immune cells to express adrenergic receptors (ADRs) [14,15,16] making them responsive to catecholamines (e.g., norepinephrine), which are either provided by the SNS [17] or produced by immune cells themselves in a context-dependent manner [18]. In RA and animal disease models of RA, there is not a unique pro- or anti-inflammatory role of the SNS, but a disease stage-dependent role. Especially, the dual function of the SNS on immune cells being pro-inflammatory in the early phase and anti-inflammatory in the late phase of arthritis has been described in several publications before [6,19,20]. This initial inflammatory role of the SNS is explained by several effects, like increased mobilization of immune cells, increased perfusion, better antigen transport and presentation, increased provision of energy, but also direct effects on immune cells, like promoting the production of pro-inflammatory cytokines by T cells [19,21], while its anti-inflammatory function in the late phase of collagen-induced arthritis (CIA) is supported by the induction of IL-10 producing regulatory B cells (Bregs) [22]. IL-10 producing Bregs are able to stop arthritis progression and thus have an anti-inflammatory effect in CIA, which has been reported in several publications [22,23,24,25]. Additional stimulation of β2-adrenergic receptors (β2-ADRs) on splenic B cells from immunized mice further enhances the anti-inflammatory potential of regulatory B cells (Bregs) in CIA by increasing IL-10 production [22].

In this study, we hypothesized that the expression of β2-ADRs on B cells, their sensitivity to a defined sympathetic stimulus, as well as downstream signaling are highly dynamic in the course of CIA, reflecting the opposite pro- and anti-inflammatory effects of sympathetic neurotransmitters in early as compared to late arthritis on a molecular level.

## 2. Materials and Methods

### 2.1. Antibodies

Primary antibodies: anti-alpha 1 adrenergic receptor (α1-ADR, Abcam, ab3462, Cambridge, UK); anti-beta 2 adrenergic receptor (β2-ADR, Abcam, ab36956); anti-beta-Arrestin-2 (β-Arrestin-2, Cell Signaling, Cambridge, UK, Clone: C16D9); anti-cyclic AMP-response element binding protein (CREB, Abcam, Clone: E306); anti-phospho-cyclic AMP-response element binding protein (pCREB, Abcam, Clone:E113); anti-extracellular regulated kinase 1/2 (ERK1/2, ThermoFisher Scientific, Waltham, MA, USA, Clone: K.913.4); anti-phospho-extracellular regulated kinase 1/2 (pERK1/2, Cell signaling, Clone: D13.14.4E; anti-G-protein-receptor-kinase-2 (GRK-2, Abcam, Clone: Y137); Interleukin-10-Phycoerythrin-conjugated (IL-10-PE, eBioscience, Frankfurt, Germany, Clone: JES5-16E3); anti-p38 mitogen activated kinase (p38 MAPK, Cell signaling, Clone: D13E1); anti-phospho-p38 mitogen activated kinase (pp38 MAPK, Cell Signaling, Clone: D3F9).

Secondary antibodies: goat anti-rabbit IgG biotin (Dako, Frankfurt, Germany; catalog number: E0432); goat anti-rabbit IgG-R-PE (Sigma-Aldrich, St. Louis, MI, USA, catalog number: P9537); Streptavidin-PE (eBioscience, ThermoFisher Scientific, catalog number: 12-4317-87).

Isotype controls: mouse IgG (Abcam, ab37355); rabbit IgG (Abcam, ab172730, Clone: EPR25A).

### 2.2. Mice

All experiments were performed with animals housed in single ventilated cages, and in accordance with German law for animal protection. Male DBA/1J mice, 6–8 weeks old were originally purchased from Elevage Janvier, Le Genest St Isle, France. Five Animals were housed in each cage and fed standard laboratory chow and water ad libitum under standard conditions of temperature and light.

### 2.3. Collagen-Induced Arthritis (CIA)

Male DBA/1J mice (6–8 weeks old) were intradermally immunized at the base of their tails with 100µL emulsion containing bovine type II collagen (2 mg/mL, Chondrex, Redmond, WA, USA) emulsified in an equal volume of complete Freund`s adjuvant (CFA; Sigma-Aldrich Munich, Taufkirchen, Germany) to induce collagen-induced arthritis (CIA). Preparation of the emulsion was done according to the manufacturer`s protocol. Mice were used at indicated time points after the initial injection. Arthritis scoring was performed by the same technical assistant, always at the same time of the day (8–9 h), to determine disease severity. Clinical scoring points were assigned to each limb to assess arthritis as described previously [26]. A score of 0 (no swelling), 1 (light swelling) or 2 (strong swelling) was determined for four toes at each paw, four paws and ankle/wrist joints. The maximum score for each mouse was 48 points. 3–6 mice per time point were used to determine the arthritis score (Table 1).

### 2.4. B Cell Isolation

Splenic mouse B cells from naïve or immunized DBA/1J mice were isolated by magnetic-activated cell sorting (MACS) via negative selection with the Pan B cell isolation Kit (Miltenyi Biotech, Bergisch Gladbach, Germany) according to the manufacturer’s protocol. The purity was controlled by flow cytometry and was 95%. Isolated B cells were used for stimulation experiments and flow cytometry stainings.

### 2.5. Stimulation of B Cells

After isolation, 1 × 10^6^ B cells were stored overnight in 99.5% PBS/0.5% FCS (starvation medium), before stimulation with the β2-ADR agonist Terbutaline (10^−6^ M) for 30 min. at 37 °C, 5% CO_2_. As control group unstimulated B cells were used. Afterwards B cells were further processed for flow cytometry stainings.

### 2.6. Flow Cytometry

B cells were fixed for 10 min. at 37 °C, 5% CO_2_ in 3% formaldehyde. Subsequently, B cells were centrifuged, resuspended in FACS buffer and stored overnight at 4 °C. B cells were incubated with mouse IgG antibody for 15 min. at RT to disable unspecific Fc receptor binding. For extracellular surface staining B cells were incubated with the primary antibody anti-β2-ADR for 1 h, followed by incubation with the PE-conjugated, secondary antibody goat anti-rabbit IgG.

For intracellular staining B cells were permeabilized with CytoPerm (BD Biosciences, Franklin Lakes, Hackensack, NJ, USA) according to the manufacturer’s protocol before incubation with the primary antibodies: anti-α1-ADR, anti-β-Arrestin-2, anti-CREB, anti-pCREB, anti-ERK1/2, anti-pERK1/2, anti-GRK-2, anti-IL-10, anti-p38 MAPK or anti-pp38 MAPK followed by 1 h incubation with the secondary biotin-conjugated goat-anti-rabbit IgG antibody. For the detection of biotin-conjugated secondary antibody, B cells were additionally incubated with the PE-conjugated streptavidin for 30 min. For all primary antibodies relevant isotype control antibodies were used. Stained B cells were analyzed by flow cytometry with the Coulter Epics XL (Beckman Coulter, Krefeld, Germany). To take into account the overlaps in flow cytometry stainings Overtone cumulative histogram subtraction was used for calculations.

### 2.7. Statistics

If not otherwise stated, data are represented as mean ± SD. Unpaired two-tailed Student *t*-test and ordinary one-way analysis of variance (ANOVA) was applied using GraphPad Prism software to calculate statistically significant differences between groups. The level of statistical significance was set at n.s. not significant, * p < 0.05, ** p < 0.01, *** p < 0.001, **** *p* < 0.0001. Each data point reflects results from B cells from one mouse. Data are pooled from two independent CIA experiments with B cells isolated from a total number of 3–6 individual mice.

## 3. Results

### 3.1. β2-ADR Positive B Cells Increase during Collagen-Induced Arthritis and Correlate with IL-10

**Figure 1:**
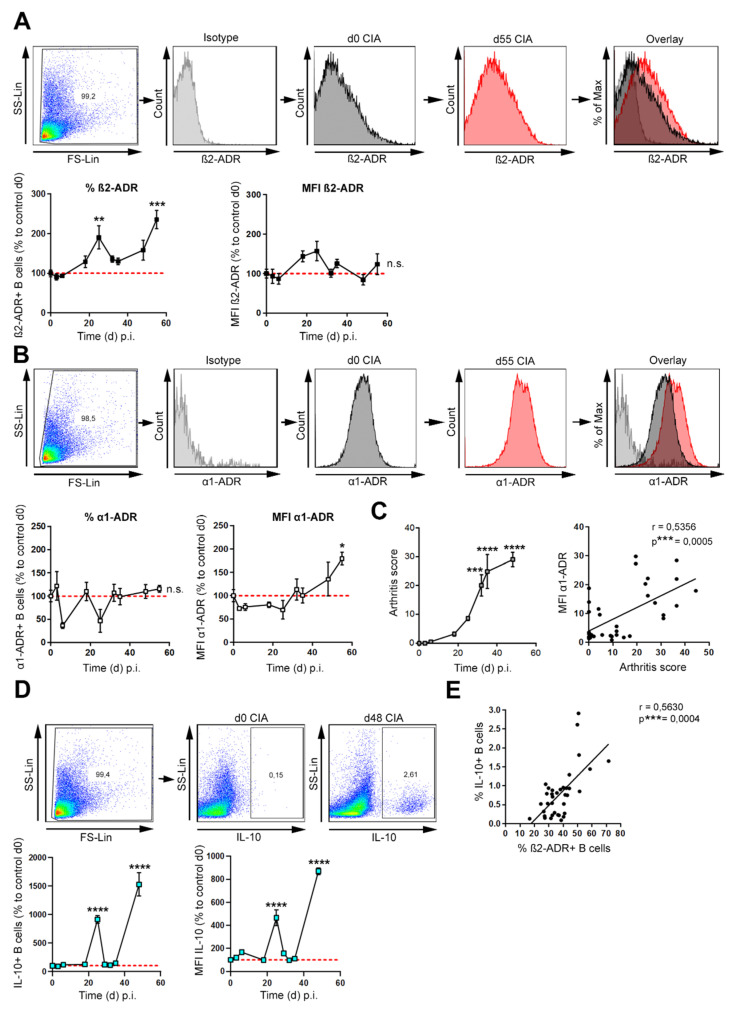
β2-ADR positive B cells increase during collagen-induced arthritis and correlate with IL-10. (**A**–**E**) DBA/1J mice were immunized with 100 µL emulsion of collagen type II and complete Freund’s adjuvant. B cells were isolated from the spleen by magnetic-activated cell sorting (MACS) at days (d) 0, 3, 6, 18, 25, 32, 35, 48 and 55 post immunization (p.i.). The frequency and mean fluorescence intensity (MFI) of β2- (**A**) and α1- (**B**) adrenergic receptors (ADRs) were measured on splenic B cells at the indicated time points (*n* = 3–6). (**C**) The arthritis score was monitored and the correlation of α1-ADRs and arthritis score was analyzed (*n* = 3–6). (**D**) The frequency and mean fluorescence intensity of IL-10-expressing B cells was analyzed by flow cytometry (*n* = 3–6). The results are shown in comparison to the mean value of d0 values from non-immunized mice (red reference line). The gating strategy is shown (**A**,**B**,**D**). (**E**) The correlation of β2-ADR and IL-10 positive B cells was quantified (*n* = 3–6). Statistical significance was determined by ordinary one-way analysis of variance (ANOVA) followed by Bonferroni post-hoc test (**A**–**D**). Continuous variables were analyzed using linear regression with r values calculated by Spearman correlation (**C**,**E**). Images are shown ± SEM. Frequency: β2-ADR: d0: 28.1% ± 6.9%; d25: 51.05% ± 11.34%; d55: 55.93% ± 7.72%. Mean: β2-ADR: d0: 1.83 ± 1.17. Frequency: α1-ADR: d0: 40.3% ± 22.9%. Mean α1-ADR: d0: 8.5 ± 6.4; d55: 25.97 ± 2.7. Frequency: IL-10: d0: 0.43% ± 0.32%; d25: 1.46% ± 0.15%; d48: 2.43% ± 0.46%; Mean: IL-10: d0: 35.98 ± 17.9; d25: 85.63 ± 17.98; d48: 160.0 ± 7.79. n.s.; not significant; * *p* < 0.5; ** *p* < 0.01; *** *p* < 0.001; **** *p* < 0.0001.

Adrenergic receptors (ADRs) are able to modulate the regulatory B cell (Breg) function by influencing the production of the anti-inflammatory cytokine IL-10, an indicator cytokine of regulatory B cells [25,27]. Especially the β2-ADR is highly expressed on B cells [28] and strongly associated with IL-10 production [22,29]. In order to investigate, whether a change in ADR expression on B cells during course of collagen-induced arthritis (CIA) might explain the dual function of sympathetic mechanisms, we analyzed receptor expression and -density of β2-ADRs and α1-ADRs at different time points of arthritis. We found a biphasic increase of β2-ADR expressing B cells during CIA with the highest expression in the late phase of arthritis, whereas there was no change in the receptor density during the entire observation period (Figure 1A). On the contrary, the percentage of α1-ADR-expressing B cells did not change during arthritis, but we observed an increase in receptor density at the late phase of arthritis (Figure 1B), which was positively correlated with disease severity (Figure 1C and Table 2 and not with duration of inflammation (Table 3). In addition, the expression of β2-ADRs was associated with α1-ADR expression (Supplementary Figure S1). To investigate whether a change in β2-ADR expression is accompanied by an increase in IL-10, we additionally analyzed the amount of IL-10-expressing B cells and the IL-10 density during course of arthritis by flow cytometry. IL-10-expressing B cells and the IL-10 density also increased in two phases during CIA, with a small increase in IL-10 in the early phase, and a profound increase in established arthritis (Figure 1D). β2-ADR and IL-10 expression in B cells positively correlated (Figure 1E). In conclusion, B cells increase β2-ADR expression and IL-10 production during CIA.

### 3.2. GRK-2 Is Downregulated during CIA, whereas β-Arrestin 2 Is Upregulated during Established CIA in B Cells

**Figure 2:**
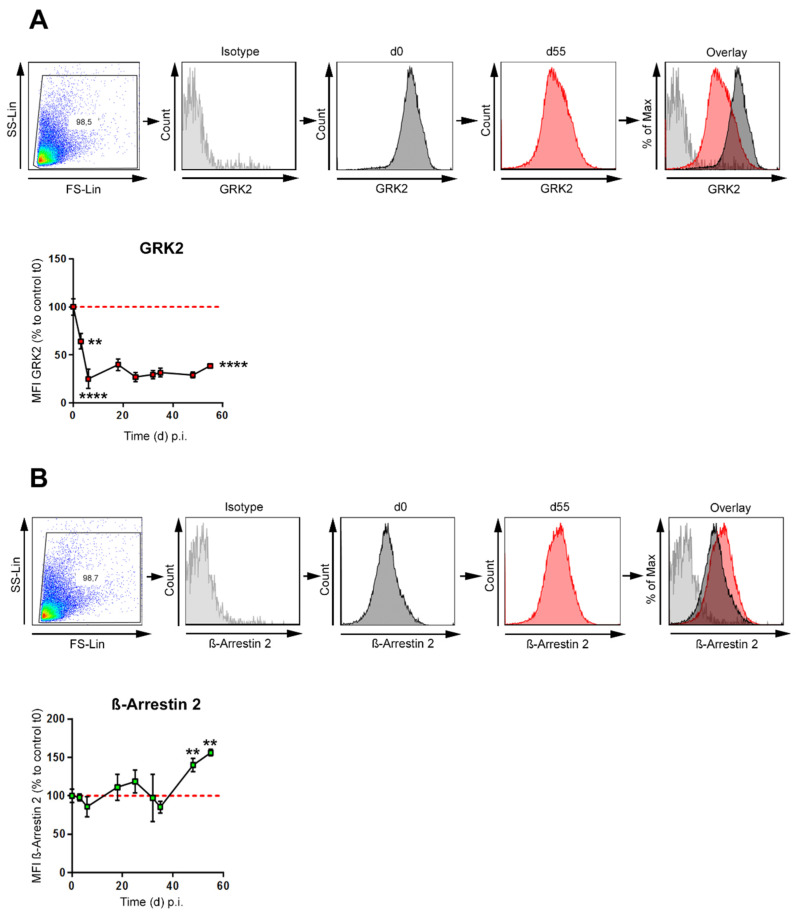
GRK-2 is downregulated during CIA, whereas β-Arrestin 2 is upregulated during established CIA in B cells. (**A**,**B**) Splenic B cells were isolated from immunized DBA/1J mice at the indicated days post immunization (p.i.). The mean fluorescence intensity (MFI) of GRK2 (**A**) and β-Arrestin 2 (**B**) was analyzed in B cells by flow cytometry (*n* = 3–6). The results are shown in comparison to the mean value of d0 values from non-immunized mice (red reference line). The gating strategy is shown (**A**,**B**). Statistical significance was determined by ordinary one-way analysis of variance (ANOVA) followed by Bonferroni post-hoc test (**A**) or Student *t*-test (**B**). Images are shown ± SEM. Mean: GRK-2: d0: 28.4 ± 22.2; d3: 4.08 ± 0.7; d6: 1.58 ± 0.61; d18: 8.76 ± 5.7; d25: 1.72 ± 0.45; d32: 6.42 ± 4.3; d35: 9.2 ± 8.4; d48: 14.6 ± 2.2; d55: 19.46 ± 1.48; β-Arrestin 2 d0: 4.35 ± 3.2; d48: 9.02 ± 1.09; d55: 11 ± 0.43. ** *p* < 0.01; **** *p* < 0.0001.

Signaling-induced phosphorylation and desensitization of G protein-coupled receptors (GPCRs) are regulated by GPCR kinase 2 (GRK2) [30]. A phosphorylation of GPCRs is associated with recruitment of β-Arrestins, in case of the β2-adrenergic receptor (β2-ADR), β-Arrestin 2 plays the major role [31]. This mediates both receptor desensitization and endocytosis [32] to terminate the GPCR signal. β2-ADRs, which belong to the GPCR family, can also signal through a G protein-independent pathway mediated by β-Arrestin 2 that leads to activation of ERK1/2 MAPKs [33]. Whether GRK2 and β-Arrestin 2 are expressed by B cells and whether there is a change in the expression during the course of collagen-induced arthritis (CIA), was investigated in B cells from immunized and non-immunized mice. GRK-2 levels profoundly decreased in B cells shortly after immunization (day 3), before any clinical symptoms are evident and remained downregulated until the end of the experiment (Figure 2A). In contrast, β-Arrestin 2 showed a small increase in the early phase and was further and significantly increased in the late phase of arthritis (Figure 2B). These data suggest that sustained β2-ADR signaling together with increased β-Arrestin 2 expression might be one mechanism to support signals via the β2-ADR and therefore anti-inflammatory B cells at late stage of arthritis.

### 3.3. β2-ADR-Stimulated Increase of pp38, pERK and pCREB Was Associated with B Cell-Derived IL-10 Production in Established Collagen-Induced Arthritis

**Figure 3:**
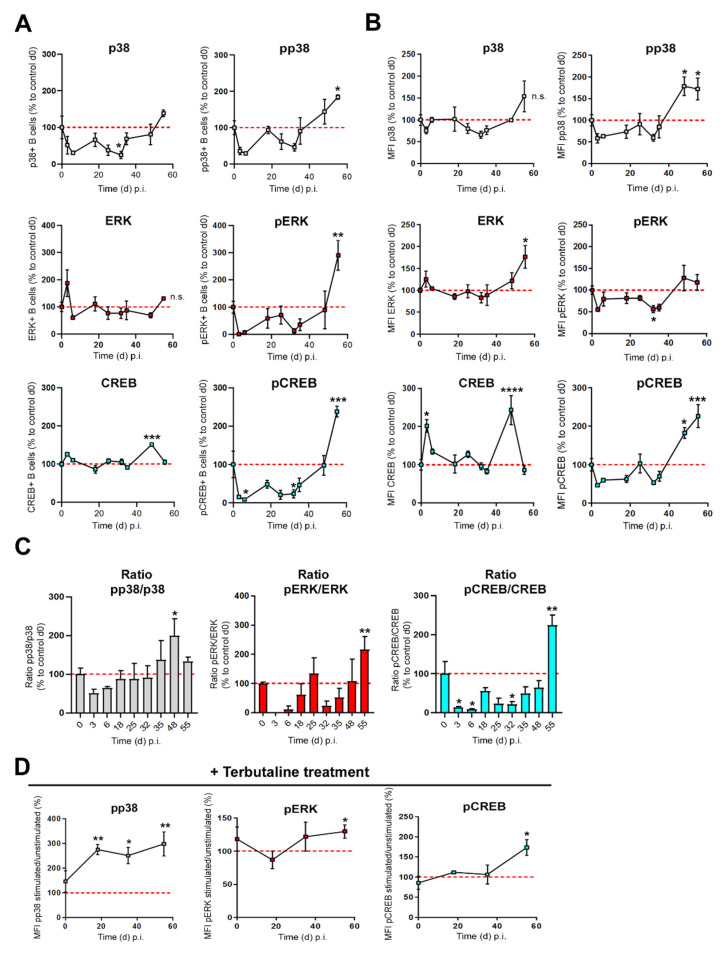
β2-ADR-stimulated increase of pp38, pERK and pCREB was associated with B cell-derived IL-10 production in established collagen-induced arthritis. (**A**–**D**) B cells from immunized DBA/1J mice were isolated from the spleen at the indicated time points and were either left unstimulated (**A**–**C**) or stimulated with the β2-adrenergic receptor (β2-ADR) agonist terbutaline (**D**). The frequency (**A**) and density (**B**) of the MAPK`s ERK 1/2 and p38 and of the transcription factor CREB were analyzed intracellularly by flow cytometry in their phosphorylated and non-phosphorylated form (**A**–**C**) (*n* = 3–6). The gating strategy is available in Supplementary Figure S2A–C. The ratio of pp38/p38, pERK/ERK and pCREB/CREB was determined (**C**). Furthermore pp38, pERK and pCREB was additionally investigated in B cells after terbutaline stimulation (**D**) (*n* = 3). The gating strategy is available in Supplementary Figure S3. The results are shown in comparison to the mean value of d0 values from non-immunized mice unstimulated (**A**–**C**) or stimulated with terbutaline (**D**) (red reference line). Statistical significance was determined by ordinary one-way analysis of variance (ANOVA) followed by Bonferroni post-hoc test ((**A**–**C**) (pCREB/CREB) and (**D**)) and Student *t*-test (pp38/p38; pERK/ERK) (**C**). Images are shown ± SEM. Frequency: p38: d0: 22.2% ± 14.4%; d32: 6.7% ± 8.1%; pp38: d0: 32.26% ± 15.7%; d55: 51.9% ± 2.52%; ERK: d0: 28.49% ± 18.96%; pERK: d0: 10.91% ± 6.1%; d55: 27.8% ± 7.4%; CREB: d0: 59.88% ± 7.79%; d48: 78.08% ± 12.59%; pCREB: d0: 23.39% ± 18.6%; d6: 2.07% ± 0.77%; d32: 5.45% ± 5.51%; d55: 53.27% ± 4.51%. Mean: p38 d0: 3.61 ± 2.5; pp38 d0: 4.5 ± 1.6; d48: 10.22 ± 1.77; d55: 9.87 ± 2.06; ERK d0: 4.0 ± 2.51; d55: 11.48 ± 2.35; pERK d0: 2.54 ± 1.17; d32: 1.05 ± 0.06; CREB d0: 1.4 ± 0.63; d3: 3.7 ± 0.42; d48: 3.1 ± 0.61; pCREB d0: 2.79 ± 1.21; d48: 6.41 ± 0.7; d55: 7.93 ± 1.48. Mean after stimulation with β2-ADR agonist terbutaline: pp38 d0: 1.31 ± 0.55; d18: 2.3 ± 0.24; d35: 2.97 ± 0.55; d55: 5.65 ± 1.29; pERK d0: 1.16 ± 0.25; d55: 2.36 ± 0.26; pCREB d0: 0.84 ± 0.23; d55: 2.24 ± 0.36. n.s.; not significant; * *p* < 0.5; ** *p* < 0.01; *** *p* < 0.001; **** *p* < 0.0001.

**Figure 4:**
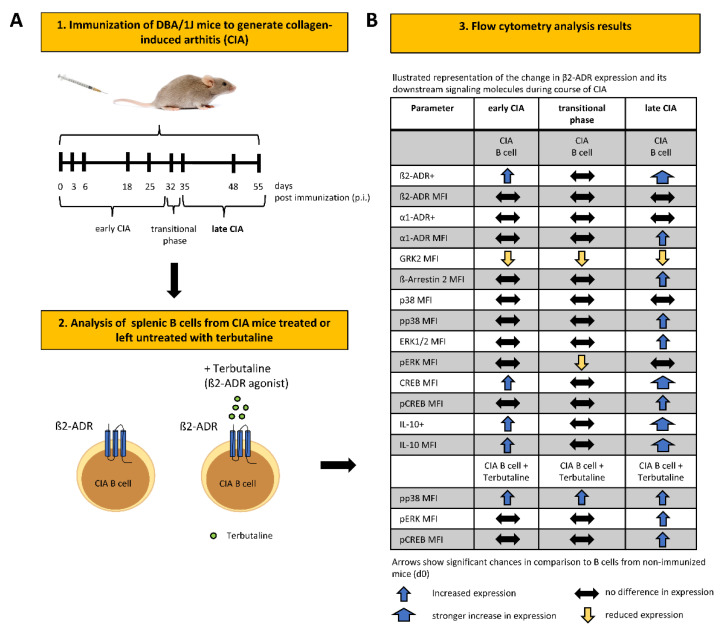
Alteration of β2-ADR expression and its downstream signaling molecules in B cells are associated with severity of inflammation during collagen-induced arthritis. (**A**): Illustrated representation of the experimental design and (**B**) of the change in β2-ADR expression and its downstream signaling molecules in B cells during the early, transitional and late phase of collagen-induced arthritis (CIA), treated or left untreated with the β2-ADR agonist terbutaline.

B cells enhance IL-10 production in vitro and in vivo following β-ADR signaling [18,22]. Activation of MAPKs such as p38 and ERK and transcription factors like CREB have been shown to be indispensable in the regulation of IL-10 by Bregs [34]. However, the molecular mechanism(s) leading to changes of IL-10 production by Bregs at different phases of CIA are poorly characterized. In order to determine which signaling pathway dominates in B cells at different stages of arthritis, the frequency (Figure 3A) and density (Figure 3B) of total p38-, ERK- and CREB proteins and their phosphorylated, active forms (pp38, pERK and pCREB) were analyzed during CIA. Moreover, the ratio of phosphorylated/non-phosphorylated proteins (Figure 3C) was investigated at different days following immunization. We found that the amount of total p38 shows a trend to decrease in the first phase of arthritis, whereas the frequency of ERK1/2-expressing B cells does not change during CIA. On the other hand, the frequency of total CREB-expressing B cells shows a trend to increase in late phase of CIA (Figure 3A). The frequency of B cells expressing the phosphorylated, active proteins pp38 and pERK increases significantly in established CIA, while frequency of pCREB-positive B cells in the spleen showed a biphasic behavior with a reduction in the early phase and an increase in late phase of CIA (Figure 3A). Regarding the fluorescence intensity (MFI) of total and phosphorylated proteins per cell we found that pp38 and pERK1/2 MAPKs started to increase in established arthritis compared to non-immunized control B cells (Figure 3B and Supplementary Figure S2A,B). In addition, we observed that total CREB was high in the splenic B cell compartment during the first few days after immunization, then decreased to basal level and increased again significantly in the late phase of arthritis (Figure 3B and Supplementary Figure S2C). In contrast, pCREB was low shortly after immunization, but also profoundly increased in the late phase of arthritis (Figure 3B and Figure 4B, Supplementary Figure S2C). The ratio of phosphorylated/nonphosphorylated protein increases for p38 and ERK MAPKs in late CIA, while the pCREB/CREB ratio shows a decrease in early and an increase in established CIA Figure 3C). To determine whether the ability of catecholamines to change phosphorylation of MAPKs p38 and ERK, as well as CREB in B cells is altered during CIA, arthritic B cells were treated with terbutaline, a β2-ADR agonist, at different time points during course of CIA. A β-adrenergic stimulus increased phosphorylation of p38 in B cells at all time points of arthritis, whereas pERK and pCREB were enhanced above the control level only in established arthritis (Figure 3D and Supplementary Figure S3). Our results suggest that IL-10-promoting signaling pathways, especially in established arthritis, are strengthend via β2-ADR mechanisms (Figure 4).

## 4. Discussion

The sympathetic nervous system (SNS) is a dynamic and changing system, varying its effects dependent on underlying inflammatory conditions and cell differentiation. In collagen-induced arthritis (CIA), stimulation of β2-ADR on B cells was able to ameliorate CIA [22]. The underlying mechanism seems to be in an increase of IL-10 positive regulatory B cells (Bregs) [22], which are capable to remarkably improve the symptoms of arthritis [22,24,35,36]. Interestingly, in adoptive transfer studies, only B cells from arthritic mice were able to ameliorate arthritis after β2-ADR stimulation [22]. Our study aimed to evaluate changes in β2-ADR signaling during CIA to explore the kind of changes that are crucial to enable B cells improving CIA after β2-ADR stimulation. Changes in β2-ADR signaling during CIA might also help to better understand the dual function of sympathetic mechanisms at different phases of arthritis [6,19]. We observed, that during CIA, B cells utilize different second messengers following adrenergic receptor stimulation depending on the phase of arthritis. It is known that the β2-ADR promotes different intracellular pathways. Data, comparing these pathways following stimulation in chronic inflammation, are lacking. To date, our data are the first comparing the different intracellular second messengers after β2-ADR stimulation in B cells during course of CIA.

During CIA, the number of β2-ADR+ B cells increases in two waves, first in the early phase (around day 25) and then again in the later phase (around day 50) of the disease (Figure 1A and Figure 4B) (a classification into different phases of arthritis was made according to [37]). Receptor density did not change during course of arthritis. The mechanism resulting in numerical increase of β2-ADR positive B cells remains unclear. Due to the known proliferative effects of cAMP and IL-1/IL-4 co-stimulation in B cells [38,39], selective proliferation of β2-ADR positive B cells during activation in secondary lymphoid organs may be one explanation. Another possibility would be the initiation of de novo synthesis of β2-ADR or a translocation of intracellular receptors in certain activated B cells by definite inflammatory mediators, which need to be identified in future studies. In addition to β2-ADR expression, various immune cells also express α1-ADRs [5].

While in primary lymphatic organs all α1-ADR subtypes are expressed, α1A- and α1B-ADRs were only detectable in cells from secondary lymphoid organs [40]. Especially for the B cells, we recently demonstrated a subgroup of α1A- and α1B-ADR positive B cells in the spleen [18]. In the current study, we found that the number of α1-ADR positive B cells did not change during CIA, but α1-ADR density increased in the late phase of arthritis (Figure 1B) and was highly associated with severity of inflammation even after control for duration of inflammation (Figure 1C and Table 2). It is known, that TNF-α and IL-1β can increase α1-ADR expression on monocytes [40,41]. Therefore, α1-ADR expression on B cells might also be induced by these cytokines, especially during periods of severe inflammation, as it is indicated by partial correlation results (Table 2). Another possible mechanism, might be the mutable regulation of adrenergic receptors as described for cardiomyocytes and THP-1 cells where, β2-ADR stimulation leads to increased α1-ADR expression [40]. In line with this assumption is the observed positive association between the number of β2-ADR and α1-ADR positive B cells in the present study. The functional consequences of α1-ADR stimulation on B cells are mostly unknown. However, in adjuvant arthritis (AA), splenocytes increased IL-10 and decreased TNF-α production after α-ADR stimulation with phentolamine [42] and clinical symptoms of CIA were ameliorated after treatment with a single dose of α- or β2-adrenergic receptor agonists. Interestingly, co-stimulation of both receptors was less effective in lowering symptoms [42]. Although this study was focused on the role of β2-ADR expression and changes in β2-ADR signaling during CIA it will be interesting to investigate in a further study if there is a crosstalk between β2- and α1-ADRs.

Our results demonstrate an increase in the number of IL-10-positive B cells and intracellular content of IL-10, especially in the late phase of CIA (Figure 1D and Figure 4B). These IL-10-producing B cells act anti-inflammatory in CIA [22]. Furthermore, IL-10 expression is increased by protein kinase A (PKA) [22], p38 and ERK MAPKs activity, as well as the transcription factor pCREB [43,44,45]. We show that, levels of pp38, pERK and pCREB in B cells are increased, dominantly in the late phase of CIA (Figure 3A,B), which is associated with an increase in IL-10-positive B cells during this phase of disease (Figure 1D). Interestingly, partial correlation analyzes (Table 2) revealed significant positive correlation between inflammation severity and these second messengers, which indicates that inflammation itself enables anti-inflammatory mechanisms. The source of adrenergic agonists in this stage of disease might be tyrosine hydroxylase (Th)+ B cells, which are able to produce catecholamines on their own [18,46], since sympathetic nerve fibers are repelled from the inflamed joint during this phase [7]. When eliminating TH+ cells using chemical sympathectomy (Sx) in the late phase an aggravation of arthritis was observed [47]. In addition, number of β2-ADR positive B cells and IL-10 positive B cells are also highly correlated and IL-10 has been shown to increase after β2-ADR stimulation [29,42].Taken together our results and literature suggests, that B cells in course of arthritis modulate the adrenergic receptor signaling profile to increase anti-inflammatory phenotypes and therefore contribute to the anti-inflammatory role of catecholamines in established CIA.

Furthermore, our results show a profound decrease in GRK2 expression shortly after induction of CIA (day 3) in most of the B cells (Figure 2A and Figure 4B). This downregulation in GRK-2 sensitizes almost all B cells to an adrenergic stimulus. This might explain why only naïve B cells from immunized mice and not from non-immunized mice are able to improve CIA, following ex vivo treatment with β2-ADR stimulating drugs [22]. Consistent with this finding, decreased levels of GRK2 during disease and increased intracellular levels of cAMP following β2-ADR stimulation are also described in RA and AA [48,49]. The early decrease in GRK2 levels might be explained by the regulation of GRK2’s via cytokines, especially IL-6 [48,49]. Other studies already demonstrated, (1) decreased GRK2 is followed by more effective β2-ADR signaling due to less β2-ADR’s phosphorylation and receptor desensitization [49,50,51,52] and (2) diminished binding of β-Arrestin 2 to the phosphorylated site of receptor, thus decreasing receptor internalization, leading to a change in intracellular signal transduction [32,33,52,53,54]. During arthritis, level of catecholaminergic messengers and density of sympathetic nerve fibers decrease in lymphatic tissues [37,55,56], hence decrease in GRK2 might serve as an early compensatory mechanism for the reduced amounts of catecholamines. Interestingly, in other important cell types (fibroblast-like synoviocytes (FLS)) in RA, inhibition of GRK2 had beneficial effects (reviewed [57]). Furthermore, inhibition of GRK2 also decreased joint damage and synovial proliferation in CIA and AA [58]. If these effects are linked to alterations in the adrenergic receptor signaling or regulation, and if GRK2 in B cells might also be valuable clinical target, needs to be determined in future studies.

The results show that β-Arrestin 2 levels, which play a predominant role in the internalization of the β2-ADRs [31], increase in B cells the late phase of arthritis (Figure 2B and Figure 4B). Our data just mirror associations and do not give mechanistic insights. In the context of current literature, the increase in β-Arrestin 2 in splenic B cells might be TNFα mediated in a p38 MAPK-dependent manner, as shown in synoviocytes [59]. β-Arrestin 2’s contribution to immune regulation is dependent on p38 MAPK [60] and NFκB [61] signaling pathways, but there is not a unique role of Arrestins contributing to inflammation or immune regulation [59,60,61,62]. β-Arrestin 1 was shown to be a positive regulator of viral-induced inflammatory cytokine production, whereas β-Arrestin 2 appeared to be a negative regulator [62,63]. In RA, FLS, TNF-α, and IL-6 production was increased by β-Arrestin 1 overexpression but was decreased by overexpression of β-Arrestin 2, indicating an Arrestin isoform-specific regulation of inflammatory responses [59,62]. In other inflammatory diseases, like chronic airway inflammation [29] and a sepsis model [64], effects of the β2-ADR were mediated through β-Arrestin 2. To our knowledge there are no data on the role of β-Arrestins in B cells during arthritis. Why β-Arrestin 2 raises only in the late phase of CIA remains unclear; however, it was shown that β-Arrestin 2 is able to support the pERK pathway [32,33,52,53,65]. Therefore, an increase in β-Arrestin 2 and its anti-inflammatory potential [62,63] following stimulation of the β2-ADR in late CIA might also contribute to the anti-inflammatory properties of B cells in this stage of disease.

Analysis of total p38 MAPK, ERK1/2, and CREB in the splenic B cell compartment during CIA showed dynamic changes, with a dominant increase of all three mediators in the late phase of CIA (Figure 3B). In addition, we observed a biphasic trend also dominant for the phosphorylated proteins with a decrease in the early and increase in the late phase of CIA, especially for pCREB (Figure 3A,B). Furthermore, our data indicate an adrenergic mechanism regulating phosphorylation of p38 and/or ERK1/2 as well as CREB in B cells. Therefore, alteration of catecholamines provided to B cells during CIA, with an initial decrease by loss of sympathetic nerve fibres and local increase of catecholamines by TH+ cells in later phases is associated with the observed biphasic timely pattern [37,47,55,56,66]. Nevertheless, all mentioned second messengers can be induced by several receptor pathways and do not exclusively reflect adrenergic signaling in general. Therefore, we also investigated the specific changes of these second messengers following β2-ADR stimulation (Figure 3D). Only the increase in pp38 following β-ADR stimulation is stable during the whole course of CIA. In contrast, β-ADR stimulation only resulted in increased pERK and pCREB in CIA B cells obtained from the late phase of arthritis. These are the first data reporting signaling pathway patterns induced by β2-ADR stimulation of B cells change, depending on the phase of CIA. Our data do not give mechanistic insights why and how the intracellular pathways change after β2-ADR stimulation. However, in the context of the current literature, the increase in pERK1/2 in the late phase could be associated with enhanced expression of β-Arrestin 2 [32,50,51,53]. However, the functional consequences of activated pERK1/2 system in B cells merits further investigation. As mentioned above, IL-10 expression is regulated by PKA, p38 MAPK, pERK MAPK and pCREB signaling [22,43,44,45,67,68,69]. Therefore, the increased number of β2-ADR+ B cells that are able to receive β2-ADR stimulation, and the increase in pp38-MAPK and pERK, which probably promote the increase in pCREB [70,71], could explain the enhanced IL-10 production after β2-ADR stimulation, especially in the late phase of CIA. Furthermore, it has recently been shown that inflammatory processes play an important role in the development of anti-inflammatory B cells, since differentiation of marginal B cells (MZ-B cells) into IL-10-producing cells was boosted under inflammatory conditions [72]. Here, our partial correlations show that it is not the duration of inflammation but the severity of inflammation that is closely associated with the observed changes in the β2-adrenergic receptor signaling pathway (Table 2 and Table 3).

A limitation of our study is, that results are obtained from flow cytometry analyzes only, whereas alternative methods, like Western Blot or gene arrays were not used to validate our findings. However, flow cytometry is highly sensitive and used in several studies for signaling pathway analysis [73,74,75]. Since we observe in part only small changes of phosphorylation in small subpopulations of cells using a highly sensitive quantitative method leads to the most reliable results as opposed to Western blot or indirect pathway determination using gene arrays, respectively.

## 5. Conclusions

In conclusion, the catecholaminergic system is a dynamic system, changing during inflammation to maintain local homeostasis. Our data will help to better understand the interaction between the SNS, B cells and the originally described dichotomy of the SNS in the early and late phases of arthritis and are useful to identify potential therapeutic targets that inhibit the production of pro-inflammatory and increase anti-inflammatory mechanisms. However, the data also show that timing of possible interventions might be of importance.

## Figures and Tables

**Table 1:** Arthitis score during course of collagen-induced arthritis.

Time (d) p.i	0	3	6	18	25	32	35	48
Arthritis score	0	0	0.5 ± 0	3.17 ± 1.6	8.6 ± 1.6	20.1 ± 8.3	24.9 ± 13.1	29 ± 5.6

**Table 2:** Partial correlations of investigated parameters with severity of inflammation controlled for duration of inflammation.

Parameter	p38	CREB	ERK	GRK2	ß2-ADR	pp38	pCREB	pERK	ß-Arrestin2	1-ADR	IL-10
Correlation	0.5	0.21	0.44	0.32	0.25	0.38	0.43	0.48	0.42	0.54	−0.244
*p*-value	0.0004	0.161	0.002	0.034	0.085	0.009	0.003	0.001	0.004	0.0002	0.141

**Table 3:** Partial correlations of investigated parameters with duration of inflammation controlled for severity of inflammation.

Parameter	p38	CREB	ERK	GRK2	β2-ADR	pp38	pCREB	pERK	β-Arrestin2	α1-ADR	IL-10
Correlation	−0.04	−0.11	−0.13	−0.37	−0.10	−0.17	−0.07	−0.08	−0.09	−0.07	0.25
*p*-value	0.785	0.471	0.383	0.016	0.492	0.242	0.664	0.589	0.531	0.675	0.121

## Data Availability

The data presented in this study are available on request from the corresponding author.

## Supplementary Materials

The following supporting information can be downloaded at: https://www.mdpi.com/article/10.3390/biomedicines10081950/s1, Figure S1: β2-ADRs correlate strongly with α1-ADRs on B cells; Figure S2: B cells show a biphasic expression of secondary messengers during CIA; Figure S3: B cells increase pp38, pERK and pCREB after β2-ADR stimulation.
